# Individual variation in local interaction rules can explain emergent patterns of spatial organization in wild baboons

**DOI:** 10.1098/rspb.2016.2243

**Published:** 2017-04-19

**Authors:** D. R. Farine, A. Strandburg-Peshkin, I. D. Couzin, T. Y. Berger-Wolf, M. C. Crofoot

**Affiliations:** 1 Department of Anthropology, University of California, 1 Shields Avenue, Davis, CA, USA; 2 Animal Behavior Graduate Group, University of California, 1 Shields Avenue, Davis, CA, USA; 3 Smithsonian Tropical Research Institute, Ancon, Panama; 4 Edward Grey Institute of Field Ornithology, Department of Zoology, University of Oxford, South Parks Road, Oxford, UK; 5 Department of Ecology and Evolutionary Biology, Princeton University, 106A Guyot Hall, Princeton, NJ, USA; 6 Department of Collective Behaviour, Max Planck Institute for Ornithology, 78464 Konstanz, Germany; 7 Chair of Biodiversity and Collective Behaviour, Department of Biology, University of Konstanz, 78464 Konstanz, Germany; 8 Department of Computer Science, University of Illinois at Chicago, 851 South Morgan Street, Chicago, IL, USA

**Keywords:** collective animal behaviour, foraging, group-living, *Papio*, social structure, within-group spatial position

## Abstract

Researchers have long noted that individuals occupy consistent spatial positions within animal groups. However, an individual's position depends not only on its own behaviour, but also on the behaviour of others. Theoretical models of collective motion suggest that global patterns of spatial assortment can arise from individual variation in local interaction rules. However, this prediction remains untested. Using high-resolution GPS tracking of members of a wild baboon troop, we identify consistent inter-individual differences in within-group spatial positioning. We then apply an algorithm that identifies what number of conspecific group members best predicts the future location of each individual (we call this the individual's *neighbourhood size*) while the troop is moving. We find clear variation in the most predictive neighbourhood size, and this variation relates to individuals' propensity to be found near the centre of their group. Using simulations, we show that having different neighbourhood sizes is a simple candidate mechanism capable of linking variation in local individual interaction rules—in this case how many conspecifics an individual interacts with—to global patterns of spatial organization, consistent with the patterns we observe in wild primates and a range of other organisms.

## 1. Introduction

For group-living animals, the position of an individual relative to its group mates can have a significant impact on its fitness [1,2]. A number of influential early biologists (including Galton [3], Williams [4] and Hamilton [5]) posited that individuals should aim to minimize their exposure to potential predators by moving into areas of the group with greater local density (often towards the centre of the group [6]). Differences in age, sex, social rank or other individual properties can generate variation in susceptibility to risk [1,7–13], and studies in wild primates [14–16] and other animals [17,18] have often found that younger, or potentially more vulnerable, group members are found closer the group's centre. However, both theoretical [19] and empirical [20–23] work suggests that peripheral positions can also be associated with higher foraging success due to having first access to resources, and can give individuals better access to personal and social information [24]. In primates, males, who are typically larger and less vulnerable, are often found towards the front of moving groups [14,25–29] where they can gain earlier access to food [21,29,30]. Patterns of spatial positioning have also been linked to dominance, with high-ranking individuals typically occupying more central locations [14,20,29,31–33]. While the risks and rewards of individuals’ spatial positions are likely to be related to where they are located, relative to the global structure of their group [1,34], the mechanisms that result in individuals having consistent spatial positions need not rely on global information, but could arise from variation in individual movement patterns (e.g. speed [35,36]) and/or variation in how individuals move relative to others [37–41].

Simulation studies have highlighted several potential mechanisms that generate differences in spatial positioning. Romey [41] first investigated how individual variation in interaction rules influenced spatial organization of groups, finding that individuals with smaller preferred nearest neighbour distances tended to end up at the centre of groups. Similarly, Couzin *et al*. [37] found that individuals with a smaller zone of repulsion (the distance below which they will be repelled from others) tended to be more central, and also found that faster individuals, or those that tended to align direction of travel more strongly with that of neighbours, tended to be located at, or close to, the front of groups. Finally, Hemelrijk [42] suggested that the tendency of high-ranking individuals to occupy central positions could be an outcome of dominance interactions: when subordinate individuals are regularly displaced, their high relative mobility may cause them to occupy peripheral positions within the group.

Despite extensive theoretical research, few empirical studies have tested whether variation in how individuals move or interact with others could drive the well-documented patterns of spatial organization in animal groups [43]. One reason is that quantifying interaction rules requires highly detailed and spatially-explicit observations of many, or all, individuals within a group [44]. Further, many of the proposed mechanisms to explain patterns of spatial positioning are likely to be difficult to differentiate using observational data alone. However, a common feature of most proposed interaction rules is that slight differences in how they are parametrized, such as the strength of the interaction, the interaction range or the number of conspecifics that an individual interacts with, can lead to variation in how individuals are positioned relative to others in their group [37,41]. Having large nearest neighbour distances, a faster movement speed or higher rates of displacing others will all result in individuals having fewer close neighbours. Simply maintaining cohesion with a smaller or larger number of neighbours is also a mechanism that could drive spatial organization in animal groups.

Despite the large number of studies linking characteristics such as age, sex, and dominance to variation in within-group positioning, we still have little understanding of the role of individual differences in driving patterns of spatial organization. Are individuals, rather than age–sex or dominance classes, found in consistent spatial positions? Are individual differences in spatial positioning linked to variation in how they move or interact with other group members? In this study, we tracked the movements of nearly all members of a wild baboon troop (*Papio anubis*) using simultaneous high-resolution (1 Hz) GPS over the course of 14 days (see electronic supplementary material, supplemental experimental procedures and figure S1) [45]. We first evaluate the degree of consistency in where individuals are positioned relative to their group mates, both in terms of their distance from the centre and their distance towards the front of the group. We then use a location prediction algorithm [46,47] that takes information about the future movement of group members to predict the location of a focal individual, and the known trajectory of that individual to estimate the prediction error. We modified this algorithm to evaluate the number of neighbours (which we call the *neighbourhood size*) that resulted in the smallest prediction error for each individual. We then tested whether an individual's neighbourhood size correlates with the patterns of intra-group positioning we observe. Finally, we implement a simple movement model, inspired by our findings, to investigate whether a mechanism based on variation in neighbourhood size can drive patterns of spatial organization in groups.

## 2. Material and methods

### (a) Data collection

Fieldwork was conducted at the Mpala Research Center (MRC) in central Kenya. From 21 to 29 July 2012, we captured 33 of 46 members of a troop of wild olive baboons (*Papio anubis*) using two arrays of individual traps (1 m^3^) baited with maize. Seven individuals were too small to be fit with a collar and were immediately released. We chemically immobilized the rest of the baboons using ketamine (15 mg kg^−1^). We estimated the age of each individual based on patterns of dental eruption and evidence of sexual maturation. Individuals with deciduous dentition were classified as juveniles. Subadult and adult males were distinguished based on their body size and the development of secondary sexual characteristics, including their mantle, musculature and canine size and morphology. Females were considered adult if they had full, permanent dentition and were parous (based on the elongation and darkening of their nipples) or showed evidenced of cycling (based on the morphology of their sexual skin). Nulliparous females that were cycling but still had one or more deciduous teeth were classified as subadult (see electronic supplementary material, table S1).

We fit 26 baboons (14 adults, 10 subadults and two large juveniles) with GPS collars (e-Obs Digital Telemetry, Gruenwald, German). One collar failed almost immediately, so analyses reported here are based on movement data from 25 individuals. Collared adults and subadults represented approximately 80% (23/29) of the total number of adults and subadults in the troop. Adults and large subadults were fit with D-cell battery collars weighing 300 g while smaller, C-cell collars (230 g) were used on small subadults and juveniles. All collars weighed less than 5% of individual body weight, and were equipped with a break-away mechanism (Advanced Telemetry Solutions, Isanti, MN, USA) that automatically detached the collar at the end of the study.

GPS collars were programmed to record location estimates continuously at 1 Hz during daylight hours (6–18 h). Sampling at this rate, C-cell collars had sufficient charge to collect data for 14 days, while D-cell collars remained active for approximately 30 days. All analyses presented here use data from the first 14 days of the study because the majority of collars remained active during this period. However, several collars failed early due to a programming bug, and so the total number of individuals tracked each day varied between 16 and 25 (electronic supplementary material, table S1). To estimate error, we conducted a test walk with a pair of GPS collars fixed 1 m apart. The average relative positional error was 0.26 m (95% CI: 0.03–0.69). We, therefore, applied limited processing to the raw data, only interpolating few missing points and removing erroneous points (see electronic supplementary material).

### (b) Determining dominance

The alpha male was determined via direct behavioural observations of his consistent priority of access to food, displacements of other individuals in the troop, and his receipt of submissive behaviour from other individuals. However, because the troop we studied was not habituated to human observers and baboons were not individually identifiable, we could not collect sufficient observations to reconstruct the rest of the dominance hierarchy through direct behavioural observation. Instead, an approximate dominance ranking for all troop members was determined by extracting approach–avoid interactions from the movement data using an automated procedure, and ranking the members of each sex separately based on an Elo score analysis. Although our inferred dominance hierarchy is consistent with our observations, our analyses also address patterns of spatial positioning related to age–sex class differences. These are a good indicator of an individual's general dominance within the troop, with all males being dominant over all females.

Candidate approach–avoid events were saved as .kml files, displayed in Google Earth, and evaluated by an observer blind to the identities of the interacting individuals (M.C.C.). Interactions that were determined to be very likely to be displacements (*N* = 290) were subsequently used to calculate each individual's Elo score [48]. Elo score-based ranking is a standard approach that computes scores based on the wins and losses among pairs of individuals. The basic principle of this ranking algorithm is that wins against competitors who are much more highly ranked result in a larger increase in score than wins against closely ranked competitors. Individuals that repeatedly win contests (in this case displace others) will gain high scores, whereas the scores of those that are displaced will drop (as a result of losing these contests). Thus, higher scores mean that individuals are more dominant (they were the ‘displacer’ more often than they were ‘displaced’). Because Elo scores are dependent on the order at which events occur (i.e. the scores are updated based on the current difference in the two interacting individuals’ values), we recalculated individuals’ Elo scores 1000 times while randomizing the order of events each time, and took the mean of these Elo scores to determine an individual's dominance rank. We note that the alpha male (2427) determined from this procedure was consistent with the individual we had identified as the alpha male through direct behavioural observation.

### (c) Statistical analysis of spatial positioning

We combined generalized linear mixed models (GLMMs) with permutation tests [49–51] to quantify (i) the stability of individuals’ spatial positions, (ii) the patterns of positioning related to age and sex and (iii) whether spatial positioning was related to dominance.

*Analysis* (*i*). We first measured the stability of individuals’ spatial positions by fitting a model of normalized distance from centroid with individual identity (ID) fitted as a random effect. Distances were normalized by computing the *z*-score (subtracting the mean distance across all group members at a given moment and dividing by the variance in these distances) to account for changes in group spread over time. From this model, we extracted the proportion of the variance that was explained by individual identities. We tested whether this value was higher than expected by chance using a permutation test where we fit the same model after randomizing individuals' identities each day. That is, each day we independently swapped the identity labels of individuals present, which enabled us to maintain the structural integrity of daily tracks, while randomizing identities across the 14 days of the study. By repeating this procedure 1000 times, we generated a distribution of per cent variance explained by identity from which we calculated a two-tailed *p*-value. This approach also enabled us to control for the changes in the presence/absence of individuals on different days arising from collar failures (see electronic supplementary material, table S1).

*Analysis* (*ii*). We tested whether there was a significant overall effect of age and sex class on the patterns of individual spatial positions. We assigned each individual to one of five age–sex classes, and added this factor as a fixed effect to the model described above (individual identity was included as a random effect in every model). To assess whether the effect of age–sex class was greater than expected by chance, we measured the variance in the coefficient values in the observed model, and compared this to the distribution of variances in coefficient values from 1000 permutations of the data. This permutation test differed from the procedure described above because we randomized the individual attributes across all days. That is, we swapped the identity and the age–sex class data together, and did this across all days together. This model maintains the consistency of GPS tracks both within and across days and the consistency of identity with age-sex class. To test whether differences existed between age–sex class (rather than overall across all classes), we performed pairwise comparisons for each combination of age–sex classes (i.e. two factors in each model) by subsetting the data where we excluded individuals from other age–sex classes. We used the same permutation test to evaluate the statistical significance of each model, but this time comparing the observed coefficient value to the distribution of coefficient values drawn from applying the same model to the 1000 permutated versions of the data [51]. Note that in these pairwise comparisons, we excluded the juvenile age–sex class because only two juveniles were present in the data.

*Analysis* (*iii*). We evaluated the association between social dominance and spatial positioning using a model of normalized distance from the centroid as a function of dominance rank. In this model, we fit dominance rank as a fixed effect and controlled for age–sex class patterns by including age–sex class as a random effect. To evaluate statistical significance, we compared the observed coefficient value of the dominance effect to a distribution drawn using the same approach as described in analysis (ii) applied to 1000 permutated versions of the data, where in each permutation we randomized the dominance rank of individuals across all days.

We tested whether our positioning results were biologically meaningful by comparing them to individual's measures of surroundedness. Surroundedness is a measure based on circular statistics that has been proposed as a robust measure of spatial centrality within groups [52]. We also evaluated the stability of individual spatial positions, as well as the effects of age–sex class and dominance along the front-to-back axis (where a position of 0 is at the centre of the group and positive values are towards the front in the direction of travel). We repeated the procedures described above, but replacing the distance from the centroid as the dependent variable in the model with distance front-to-back from the centroid. Distances were normalized into *z*-scores to account for variation in group spread.

### (d) Determining neighbourhood size

To quantify variation among individuals in their neighbourhood sizes, we modified a framework based on location prediction to find the number of neighbours that provide the most accurate predictions [46,47]. The basic framework works as follows (see also electronic supplementary material, figure S2):

(1) For each individual, we start by randomly selecting 1000 observations (initial times) in the data.
(2) We then identify the individual's *k* nearest neighbours at each initial time.
(3) Using the GPS data from the same set of *k* nearest neighbours identified in step 2, we calculate their mean location (centroid) each second (time lag) for up to 600 s after the original observation time.
(4) We use this centroid to predict the location of the focal individual at each second, and calculate the prediction error as the distance between this location and the actual location from the GPS data recorded for that individual.
(5) We then find the optimal value of *k* (range 1–24) that generates the lowest mean prediction error at each time lag. We define an individual's *neighbourhood size* as the mean of these optimal values of *k* across all time lags.

Note that within each replicate, the centroid used for prediction is calculated using the same set of focal individual's *k* nearest neighbours (those that were the individual's nearest neighbours at the initial time).

### (e) Determining the relationship between neighbourhood size and position in the group

We first tested whether there was a relationship between an individual's *neighbourhood size* (defined above) and its mean distance from the troop centroid across all observed data by computing the Spearman rank correlation between these two variables.

We also tested whether *neighbourhood size* itself could represent an artefact of individuals having different positions—that is whether being at the centre itself (regardless of by what mechanism this central position is achieved) leads to a higher estimated neighbourhood size, thus biasing the data towards a higher *k*. For each unique prediction of an individual from a given start time, we recorded the best supported neighbourhood size (*k*). We then compared these values of *k* to the focal individual's *current* distance from the centroid at the time the prediction was made (*t_f_*). We computed the mean value of *k* for each individual from the instances when it occupied a position within a certain range of distances from the troop centroid. We then tested whether there was a relationship between an individual's neighbourhood size and its *mean* distance from the group centroid, while controlling for its *current* distance from the group centroid at the time of the prediction. To account for differences in group spread, we also performed this analysis using each individual's current *ranked* distance rather than its absolute distance from the centroid.

### (f) Simulation model of spatial positions arising from neighbourhood size variation

We constructed a simple one-dimensional model to assess the impact of variation in neighbourhood size on emergent spatial patterns. The model is initialized with *N* individuals located at random positions, which are drawn from a uniform distribution ranging between 0 and 1. Each individual is assigned a neighbourhood size *k*, which determines how many nearest neighbours it interacts with. At each time step, a focal individual is selected at random to move. With a probability *p*, it moves a distance *d* in a random direction, where *d* is drawn from a normal distribution with mean 0 and standard deviation *σ*. With probability 1 – *p* it moves a distance *s* towards the centroid of its *k* nearest neighbours, unless *s* is greater than this distance, in which case it simply moves to the centroid of its *k* nearest neighbours. This process is repeated *t* times, and the final distance of all individuals to the *group* centroid (note the distinction between the group centroid and the centroid of the *k* nearest neighbours) is recorded. In the results presented here, we ran 1000 simulations with *N* = 25 individuals and set the distribution of *k* values to be equal to that observed in the data. Each simulation consisted of 100 samples (replicates of the model taken using a single group). We set the other parameters as follows: *p* = 0.5, *σ* = 0.01, *s* = 0.1 and *t* = 1000.

We also implemented a similar model in two dimensions, where individuals are initially placed uniformly at random within a circle of radius 1, and at each time step an individual moves towards the centre of its *k* nearest neighbours (with probability 1 − *p*) or, with probability *p*, it takes a random step in both the *x*- and *y*-directions (with the step length for each dimension determined as in the one-dimensional model). We confirmed that this two-dimensional model yielded the same negative relationship between an individual's value of *k* and its final distance from the group centroid as seen in the one-dimensional case. In both one- and two-dimensional models, we investigated a range of parameter values and noted that while the quantitative results change, this negative relationship is retained.

## 3. Results

### (a) Are individual characteristics associated with spatial positioning patterns?

**Figure 1.**
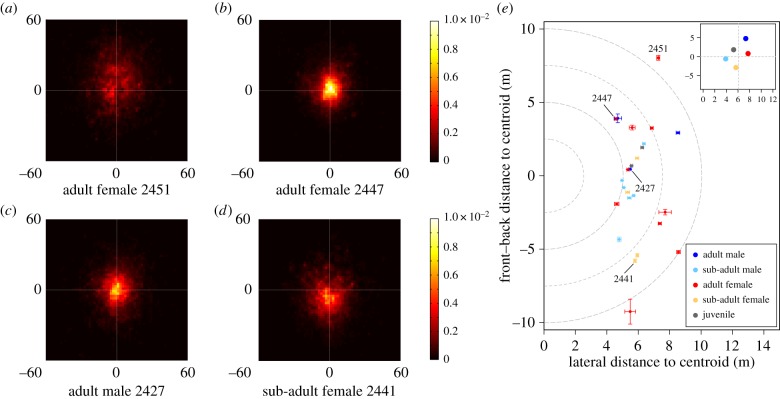
Individuals exhibited markedly different patterns of spatial positioning within the group. (*a–d*) Histograms showing the probability of occupying a given position relative to the group (colour) for four different individuals. The origin of each plot indicates the troop centroid (white point), and the positive *y*-axis points in the direction of troop movement. Individuals had consistent positioning patterns that ranged from peripheral (*a*) to central (*b*,*c*) and from front (*a*) to back (*d*). Differences in spatial position were consistent across days (electronic supplementary material, figure S3), including mean front/back and lateral position (shown for all individuals in (*e*), bars are standard errors of the mean). Inset shows the age–sex class averages. Class-level results indicate that adults typically occupied more frontal and lateral positions, while subadults and juveniles were typically more central and found towards the back of the troop. (Online version in colour.)

Individuals varied consistently in their distances from the centre of the group. We found that individual identity explained 18.0% (*p* < 0.001; electronic supplementary material, table S2) of the variance in distance from the centre of the group (analysis (i), figure 1; electronic supplementary material, figure S3), over the course of our observation period. Subadults and juveniles were more centrally located than adults, and male subadults were more central than female subadults. However, controlling for individual identity, the observed variance in coefficient values across age–sex classes was not significantly larger than expected by chance, possibly due to the small number of individuals in each age–sex class (analysis (ii), see electronic supplementary material, table S3 for the full GLMM model outputs). When comparing each pair of age–sex classes, subadult males were found to be significantly more central than other age–sex class groups (electronic supplementary material, table S4). We also found that dominance (i.e. small rank number) within each age–sex class was associated with a significantly smaller distance from the group centroid than would be expected by chance (analysis (iii), *β* ± s.e. = 0.04 ± 0.01, *p* = 0.048; electronic supplementary material, table S8). Individuals in central positions also tended to be more surrounded by their troop mates (electronic supplementary material, figure S4), meaning that their troop mates are distributed more equally in space around them.

Baboons also showed consistent individual differences in their front–back positions within the troop, with individual identity accounting for 27.8% (*p* < 0.001; electronic supplementary material, table S5) of the variance across our minute-by-minute observations. While the observed variance in the coefficient values for each age–sex class did not differ significantly from what would be expected by chance (*p* = 0.424, see electronic supplementary material, table S6), pairwise comparisons confirm that adult males occupied positions significantly more towards the front than subadults (both males and females; electronic supplementary material, table S7). Within each age–sex class, high-ranking baboons also tended to be found more in front of the group centroid than subordinates, however, this result was not statistically significant (*β* ± s.e. = −0.01 ± 0.001, *p* = 0.106; electronic supplementary material, table S9), and this was not the case for the alpha male (figure 1*e*).

### (b) Can global differences in within-group spatial positioning emerge from variation in local interaction rules?

**Figure 2.**
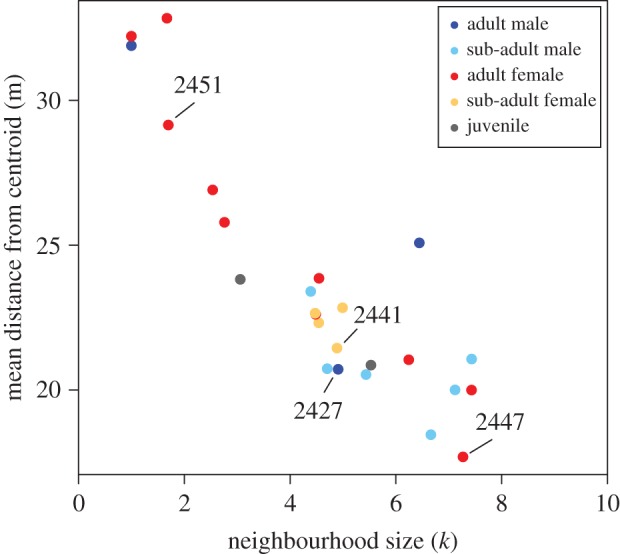
Individuals with a large neighbourhood size are typically found closer to the troop centroid. Each point represents an individual's mean distance from the troop centroid (figure 1) and its neighbourhood size (mean value of *k* that generates the most accurate location prediction for that individual across all time lags). The four individuals shown in figure 1 are also labelled here (text labels). (Online version in colour.)

**Figure 3.**
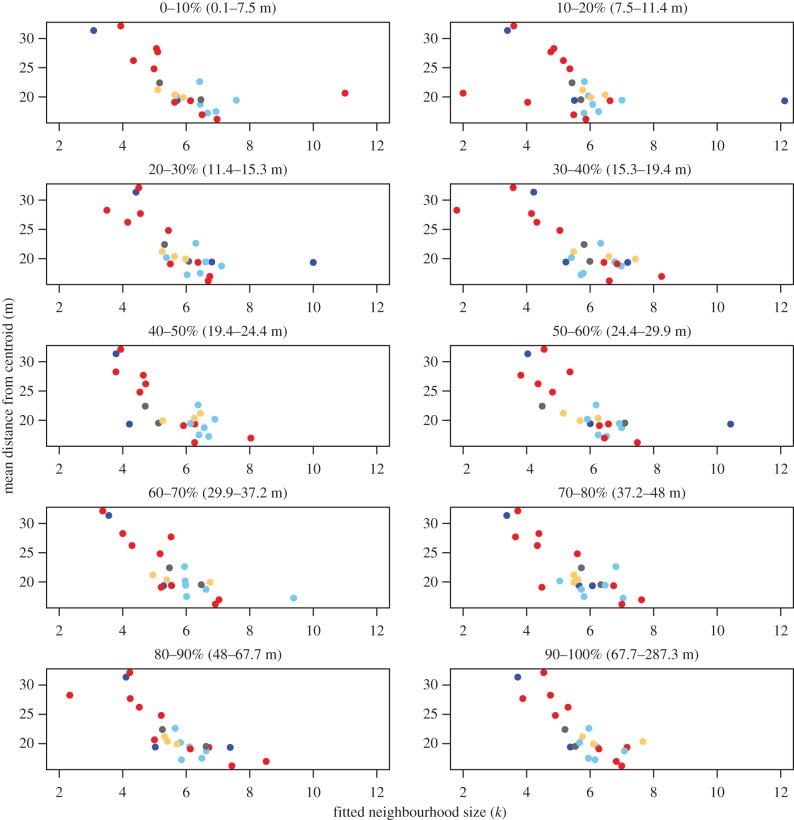
The relationship between an individual's neighbourhood size and its mean distance from the troop centroid persists regardless of its distance from the troop centroid at the time of the prediction. Each plot shows the mean optimal value of *k* for each individual (*x*-axis), computed based only on data from when the individuals were located within a specified range of distances from the troop centroid (top labels). Distances from the centroid are binned based on 10% quantiles (i.e. the top left panel represents data from each baboon when they were in the lowest 10% of distances from centroid). In all cases, data show a negative relationship, indicating that individuals that are found on average closer to the centroid have a larger neighbourhood size regardless of where they are *currently*. This negative relationship is also maintained if *ranked* distances from the centroid are considered rather than absolute differences (electronic supplementary material, figure S6). In addition, fitted neighbourhood sizes are relatively consistent within an individual regardless of its current location (see also electronic supplementary material, figure S5). (Online version in colour.)

**Figure 4.**
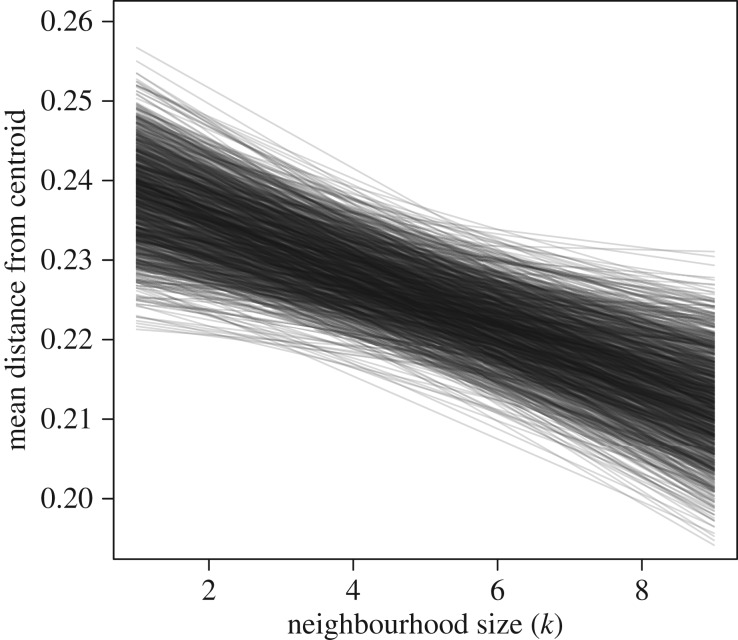
Results from a simple simulation replicate the relationship observed in the data. After simulating movement of 25 individuals in one-dimensional space using the same distribution of neighbourhood sizes as observed in the data (figure 2), those individuals with a larger neighbourhood size were typically found closer to the group's centroid. Each simulation consisted of 1000 samples, and we recorded each individual's mean distance from centroid across all samples. Each line represents the relationship between neighbourhood size and distance from centroid from a single simulation. The units of distance are arbitrary. Similar results were obtained using a two-dimensional model (electronic supplementary material, figure S8).

Individuals varied in their neighbourhood sizes, with the most accurate predictions coming from *k* values that ranged from 1 to 8 neighbours. We note that the real number could be slightly higher given that 20% of the adults and subadult members of the troop were not fitted with collars. Despite this potential limitation, we found a clear relationship between an individual's neighbourhood size and its mean distance from the group centroid (figure 2). Those with larger neighbourhood sizes tended to be observed closer to the centre of the group (Spearman's rank correlation = −0.77, *p* < 0.001). Individual baboons appear to have relatively consistent neighbourhood sizes regardless of the position they currently occupy (electronic supplementary material, figure S5), and the negative relationship between individuals’ fitted *k* values and their mean distance from the centroid is maintained across all distance ranges (figure 3). Finally, simulations of our toy model demonstrate that individuals with higher values of *k* do consistently end up closer to the centre of the group than individuals with lower values of *k* (figure 4; electronic supplementary material, figure S8).

## 4. Discussion

Revealing the mechanisms that determine how animals form and maintain groups is fundamental to understanding the evolutionary dynamics of social organization [37,53]. The study of the spatial organization of baboon troops in particular has had a long history, from the original proposal of DeVore & Washburn [9] that adult males surround juveniles to protect them from predators, to more recent theoretical work that has emphasized that positioning patterns may arise through individuals optimizing the trade-off between predation risk and foraging competition [19,54–56]. In our study, we observed that although individual positioning within baboon troops is highly dynamic, individuals showed consistent patterns of within-group spatial positioning, with their distributions of positions, relative to the group's centre and direction, being consistent across days. As has been reported in previous work on primates [14–16] and other animals [57], individual differences in spatial positioning were found to be associated with both age and sex. We found that subadults (in particular subadult males) were generally positioned closer to the group centre than adults, and were consequently more surrounded by troop mates. This observation is broadly consistent with the hypothesis that more vulnerable members of the group should be more sensitive to predation risk, and thus prefer to occupy positions in which they are more surrounded by their conspecifics. We also found that within a given age–sex class, dominant individuals tended to occupy more central positions within the group than subordinates. That higher-ranked animals were more central is consistent with previous empirical results from a range of taxa [21,22,31,42,57–59], and with the theory that dominant individuals can better afford central positions as they can displace competitors from food sources [6,42].

While many studies report consistent inter-individual, or class-level, differences in spatial positioning within groups, few address the underlying mechanisms that could drive such patterns [37]. Differences in position could easily result from some individuals being more strongly attracted to (or repulsed by) the centre of the group. However, this would require animals to maintain a global overview of where all, or most other, group members are positioned, a feat that becomes more challenging when group sizes exceed a few individuals or in habitats that limit sensory perception. Theoretical models of collective motion have repeatedly shown that group-level coordination can emerge from individuals responding only to nearby neighbours (i.e. following simple local interaction rules) without any need for global awareness [37,60,61]. For example, in simulation models where individuals are programmed with different parameters for their repulsion rules, those using rules that lead to smaller nearest neighbour distances tend to occupy more central positions [37,41].

Our results are consistent with a simple local mechanism that could generate the emergent patterns of spatial positioning that we observe—individuals that maintain cohesion with a larger number of neighbours inherently end up at the centre of the group. The reason for this result is simple—the centroid of a greater number of an individual's nearest neighbours will on average be closer to the centre of the group [62]. Thus, the patterns of consistent individual positioning that have been widely observed in animal aggregations may not require individuals to have global information about their group. Instead, differences in position can emerge from a simple geometrical truism, highlighting the potential generality of neighbourhood size as a mechanism underlying spatial organization in baboons and other animal aggregations.

Our analysis also allows us to reject the possibility that the negative relationship we observe between individuals’ neighbourhood sizes and their mean distance from the troop centroid is purely an artefact of averaging, whereby individuals who spent more time near the centre have more data from these high-*k* instances, and consequently, appeared to have a larger neighbourhood size on average. If this were the case, we would then expect those same individuals to have low *k* values on the rarer occasions when they are found near the periphery of the group. By incorporating only a narrow range of individuals’ *current* distances from the centroid at the time of prediction, essentially controlling for this potential bias, we found that the negative relationship between individuals’ *k* values and their mean distance from the centroid was maintained. That is, individuals with higher *k* values have higher *k* values regardless of where they are currently positioned in the group. These results suggest that individual's neighbourhood size is an individual-level characteristic, and not a by-product of its current spatial position.

We also tested whether differences in movement alone, as opposed to movement resulting from interactions, could explain patterns of spatial positioning. In homing pigeons (*Columbia livia*), individual differences in speed have been shown to explain many of the patterns of positioning and leadership [35,36]. Similarly, in groups with stop–go movements, commonly found in terrestrial organisms, more active individuals could be in peripheral positions more often if their high activity means that they keep reaching the group edge. However, we found no relationship between the per cent of time that individuals spent moving and their distance from the centroid (electronic supplementary material, figure S7). Thus, our results are not directly explained by simple differences in movement behaviour.

By ruling out alternative explanations, our study lends credence to the hypothesis that variation in local interaction rules drives the global patterns of spatial organization frequently observed in primates and other animals. Baboons that maintain cohesion with a larger set of neighbours could be drawn towards the centre of the group without any need for information about the locations and configuration of all troop mates. While this local mechanism provides a plausible explanation for how patterns of positioning relative to the centre of the group can emerge, it does not address the patterns of front-to-back positioning. Theoretical models have shown that differences in local interactions can lead to self-sorting along the front–back axis [37], and frontal positions have been linked to differences in individual motivation to gain preferential access to food sources [19]. Any such factor adding a greater force (or speed) for some individual in the direction of movement could lead to variation in front–back patterns of positioning.

Our study does not reject the hypothesis that variation in spatial positioning is linked to trade-offs in costs/benefits of having different spatial positions. Instead, we suggest that variation in neighbourhood size could be a simple mechanism on which selection arising from cost/benefit trade-offs can act, as neighbourhood sizes could be closely linked to competitive environments or safety from predators. For example, following the Hemelrijk dominance hypothesis [42], subordinates could be more peripheral because having larger neighbourhood sizes could lead to more encounters, and agonistic interactions, with central dominants, thus driving the evolution of smaller neighbourhood sizes in subordinates. However, in contrast to that hypothesis, we only observed a within-class effect of dominance on centrality, and subordinate males were consistently more central than most adult males despite the fact that subordinate males should be most impacted by dominance interactions.

Our models reveal a significant portion of variation in positioning determined by individual identity. One factor that we could not address is the relatedness among individuals. Individuals with more kin, or those that are more related to others, could have stronger tendencies to remain closer to others. Such patterns could drive some of the individual-level differences in neighbourhood sizes we observed, and would be particularly strongly defined among adults. In fact, our data does suggest that there is greater individual variation in spatial positioning among adults compared to subadults (electronic supplementary material, figure S4). Hence, such a mechanism warrants further investigation in a troop where more background information is available.

Elucidating the mechanism driving variation in within-group positioning is important, as it is this mechanism upon which selection arising from ecological conditions can act. Identifying the individual rules underlying group-level patterns is a challenging task, as many rule sets can give rise to similar aggregate patterns, making it difficult if not impossible to definitively pin down this mechanism. However, here we present a simple mechanism by which individuals could achieve consistent positions within groups, and show that such a mechanism is consistent with our data. Given its simplicity, individual variation in neighbourhood size (being attracted to, and maintaining cohesion with, varying numbers of individuals) is a plausible mechanism that could be responsible for shaping the spatial organization of many animal groups.

## Supplementary Material

Supplemental Experimental Procedures

## Supplementary Material

Supplemental Figures and Tables

## Data accessibility

Data are deposited at www.datarepository.movebank.org/ (doi:10.5441/001/1.kn0816jn) [45].
