# Supplementary material for: Individual variation in local interaction rules can explain emergent patterns of spatial organization in wild baboons
Source: Proc Biol Sci. 2017 Apr 19;284(1853):20162243. doi: 10.1098/rspb.2016.2243 (PMC5413915; doi:10.1098/rspb.2016.2243)
Supplement: Supplemental Experimental Procedures [file rspb20162243supp1.pdf]

SUPPLEMENTAL EXPERIMENTAL PROCEDURES

For : Farine *et al*.

Individual variation in local interaction rules can explain emergent patterns of spatial organisation in wild baboons

Data processing

GPS devices were capable of logging data at a resolution of 1 Hz (1 point per second). We did not apply any smoothing to the data downloaded from the devices. Occasionally, some devices failed to log one or a few points. In those cases, we linearly interpolated missing points based on the existing data around that point from the same device. Randomly selecting 5000 points from the data suggested that the expected error from linear interpolation was significantly less than the GPS error (< 0.2m difference between the observed and interpolated points in cases of a single missing data point). We also found a few cases of unrealistic speeds in the data, where an individual trajectory moved a large distance off a stable trajectory for a single second before resuming the trajectory. We used the same linear interpolation algorithm to identify the 99.9th percentile error (equivalent to speeds in excess of 3.56 m/sec) between the interpolated and observed GPS points, and replaced these points with the interpolated data instead. In total, 7.4% of the data used was interpolated, most of which (7.2%) were replacements of missing values.

To assess individual position within the group, we computed each individual's position relative to the troop centroid and troop heading by projecting their locations into the reference frame of the troop. The reference frame of the troop refers to the x-y position of individuals relative to the troop centroid (where the centroid is defined as the mean x-y location across all individuals at a given moment in time). We calculated each individual's distance from the troop centroid at each point in time (once per minute). Because the spread of the troop

varied across different times, we transformed these data into z-scores by subtracting the mean and dividing by the standard deviation. These z-scores were used in all subsequent statistical analyses.

We also rotated the reference frame of the troop relative to the direction of group travel (see Figure S1) to calculate individuals' positions in front or behind the center of the group (the y-axis in the troop reference frame). To avoid the effects of "jitter" that occur when the troop is relatively stationary, which cause the heading to point in random directions, the troop heading was determined by spatially discretizing the trajectory of the troop centroid. Spatial discretization is a method of breaking up a trajectory into approximately equal-length steps. Starting from an initial focal point, the first point in the trajectory that falls outside a radius of 10 meters from this initial point is computed. This next point then becomes the focal point, and the process repeats.
