# Supplementary material for: Individual variation in local interaction rules can explain emergent patterns of spatial organization in wild baboons
Source: Proc Biol Sci. 2017 Apr 19;284(1853):20162243. doi: 10.1098/rspb.2016.2243 (PMC5413915; doi:10.1098/rspb.2016.2243)
Supplement: Supplemental Figures and Tables [file rspb20162243supp2.pdf]

SUPPLEMENTAL FIGURES AND TABLES

For : Farine *et al*.

Individual variation in local interaction rules can explain emergent patterns of spatial organisation in wild baboons

SUPPLEMENTAL FIGURES

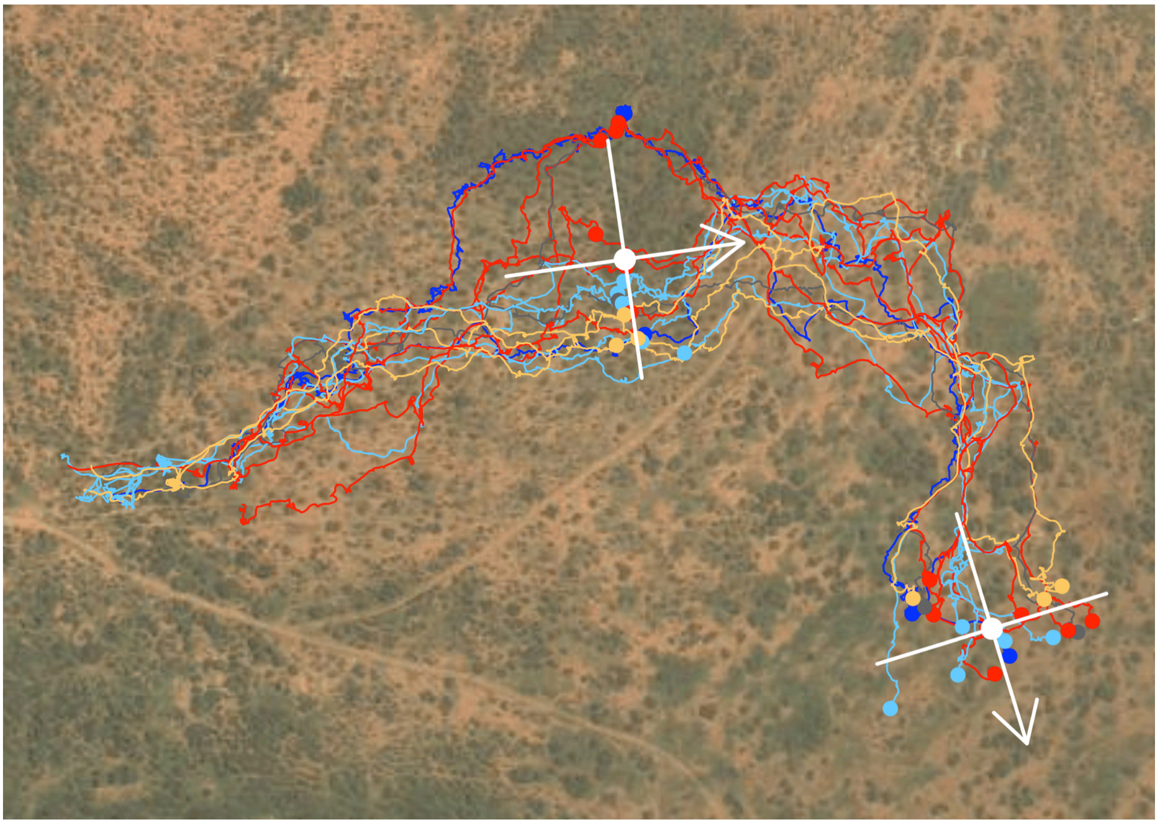

Figure S1: Computing individual positions relative to the frame of reference of the group. The group centroid (white point) at each time is defined as the mean location of all individuals at that time. The heading (white arrow) is determined by the direction of motion of the group centroid, using spatial discretisation to avoid local jitter. Color of points (individuals) and lines (their trajectories) represent different age-sex classes (see legend in Figure 1).

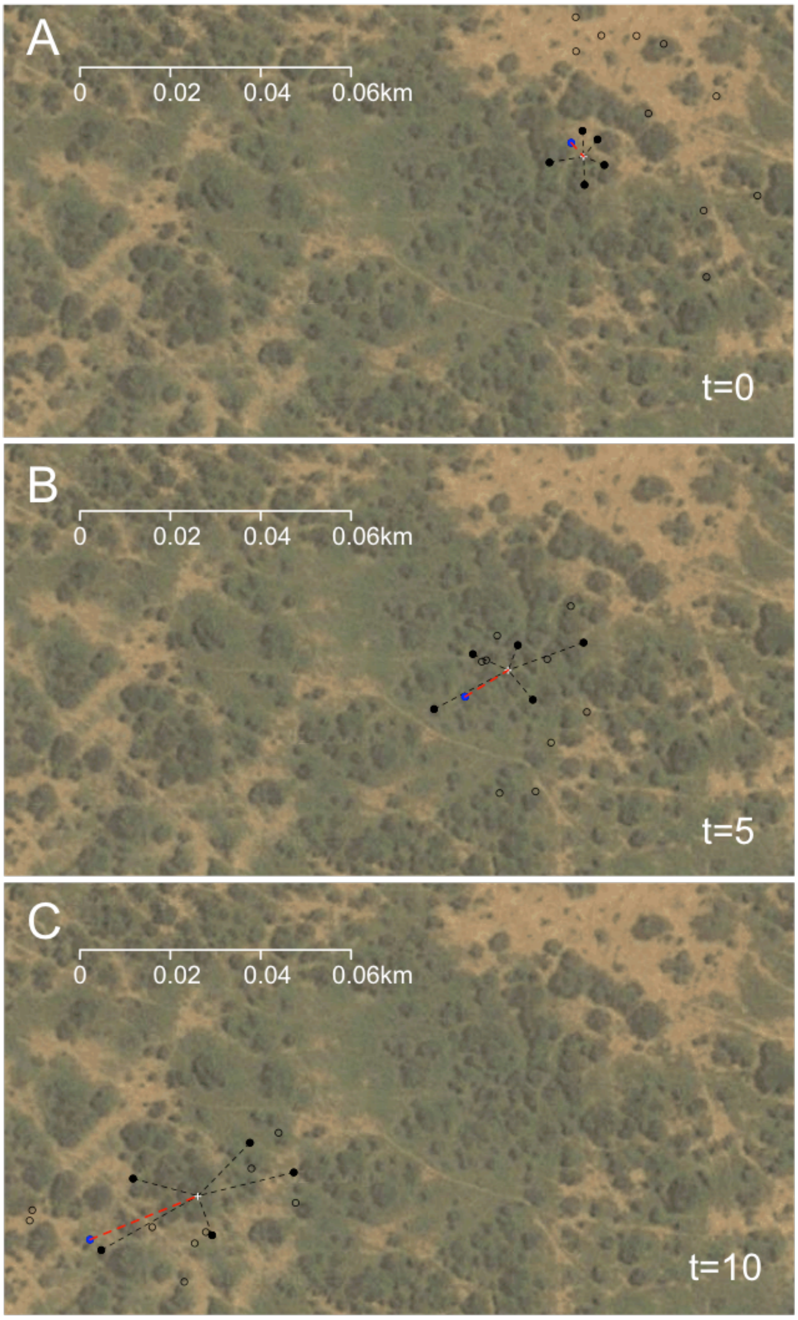

Figure S2: Example of location prediction based on *k* = 5 nearest neighbours. (A) The neighbours (black points) of a focal individual (blue point) are determined at an initial time (t=0). The location of the focal individual is then predicted using the centroid of these same neighbours (white cross) at a later time, here after 5 minutes (B) and 10 minutes (C). The error (red line) is defined as the distance between the predicted location and the actual location of the focal individual at each time. Open points represent the other troop members' locations.

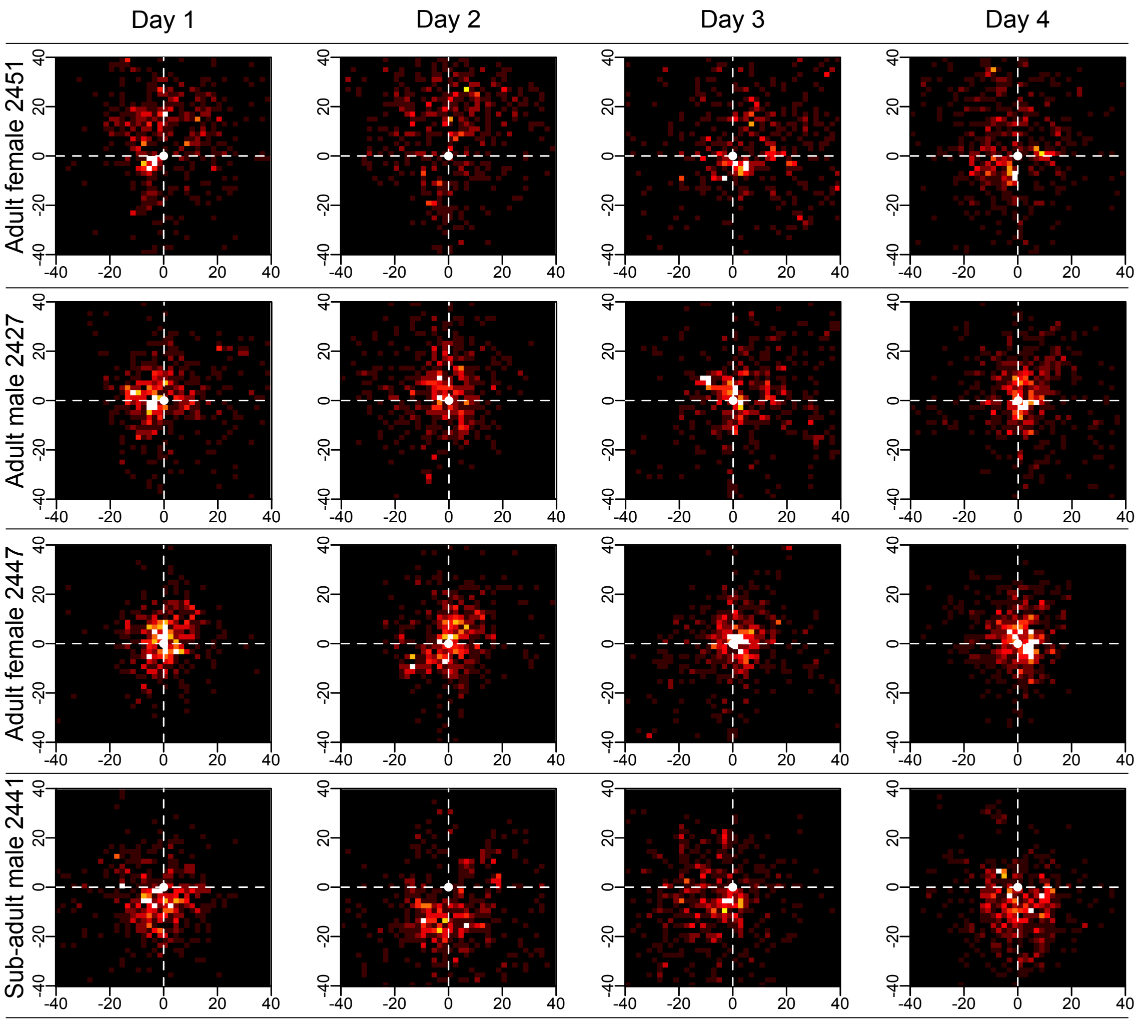

Figure S3: Individuals show consistent patterns of within-group spatial positioning across days. Histograms show the probability of occupying a given position relative to the group (color) for the four different individuals from Figure 1 (each row represents a different individual) over the first four days (each column represents a different day). The origin of each plot indicates the troop centroid (white point), and the positive y axis points in the direction of troop movement.

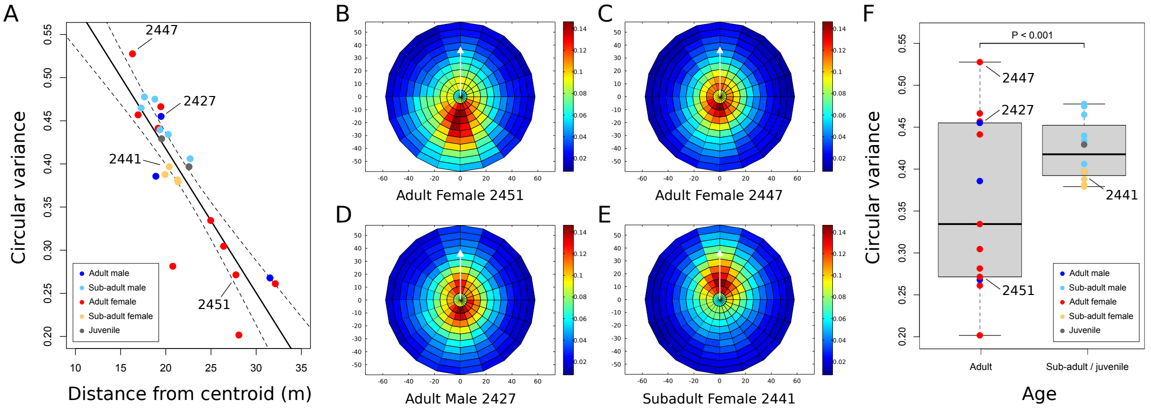

Figure S4: Different positions within the group entail different levels of surroundedness. (A) Individuals were significantly more surrounded by conspecifics when they occupied positions closer to the troop centroid. (P<0.001, lines indicate the fit of a linear model with 95% confidence intervals, points show the mean values for each individual). Surroundedness was measured using the circular variance of the vectors pointing from the focal individual to all other individuals (higher circular variance indicates higher surroundedness). (B-E) Individuals varied in how well surrounded they were by conspecifics. Each plot is centered at an individual's current position, with the positive y axis representing the direction of troop movement. Color shows the probability of at least one conspecific being present at each given location relative to the focal individual. Four example individuals are shown (the same individuals as shown in Figure 1). (E) Adults experienced significantly lower levels of surroundedness (circular variance) than did sub-adults and juveniles (β±SE=0.064±0.031, P<0.001, Figure 3E). In addition, all sub-adult males were more surrounded than all sub-adult females (β±SE=0.063±0.014, P<0.001).

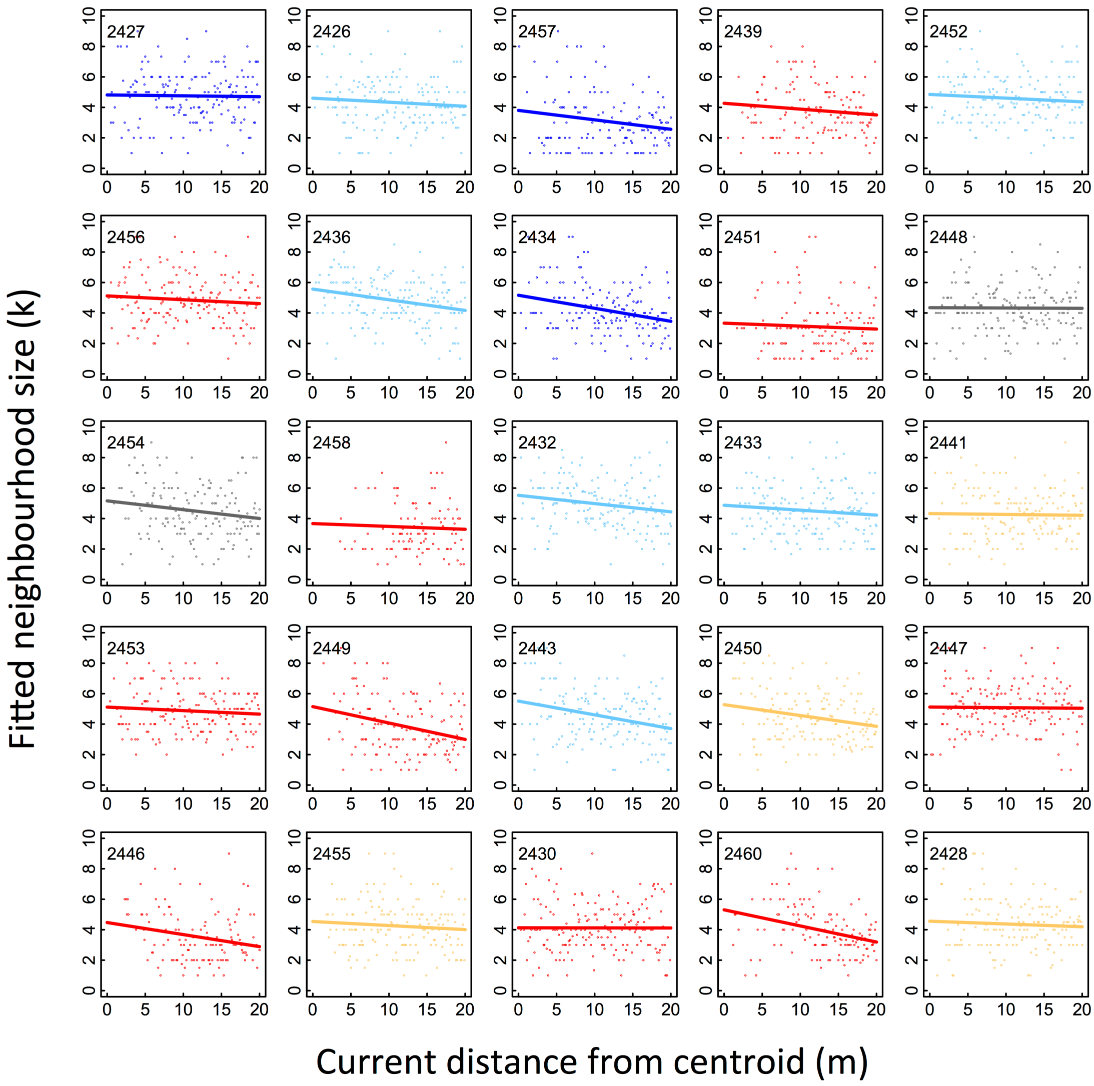

Figure S5: Relationship between final position at the end of a prediction and the most predictive neighbourhood size for each individual. Individuals on average had no, or a weakly negative relationship between neighbourhood size and their final position in the group for a given prediction period, suggesting that neighbourhood size is not an artifact of individuals' current position, but rather is more likely to be an individual-level trait. Each point represents a single prediction and lines represent linear fits.

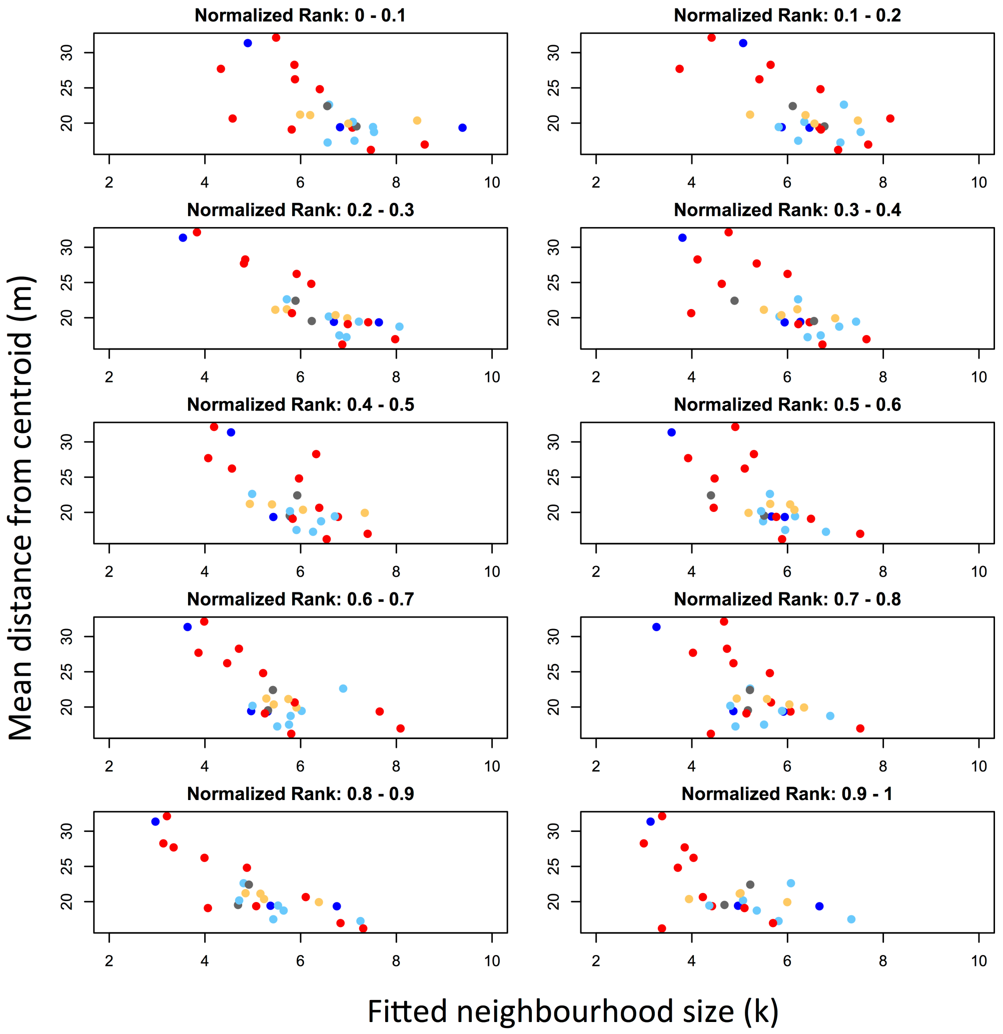

Figure S6: The relationship between an individual's neighbourhood size and its mean distance from the troop centroid persists regardless of its current ranked distance from the troop centroid at the time of the prediction. Plots are the same as in Figure 5, but in this case data are binned based on the individual's current *normalized ranked* distance, rather than its raw distance from the centroid, in order to account for variation in group spread (each panel represents a different bin). Normalized ranks were computed by calculating the distance from the centroid of all tracked baboons at the time of the prediction (t_f), ranking these distances, and normalizing them between 0 and 1 (where 0 represents the closest baboon to the center and 1 represents the farthest baboon). Normalization was performed to account for the changing number of baboons tracked at any particular time.

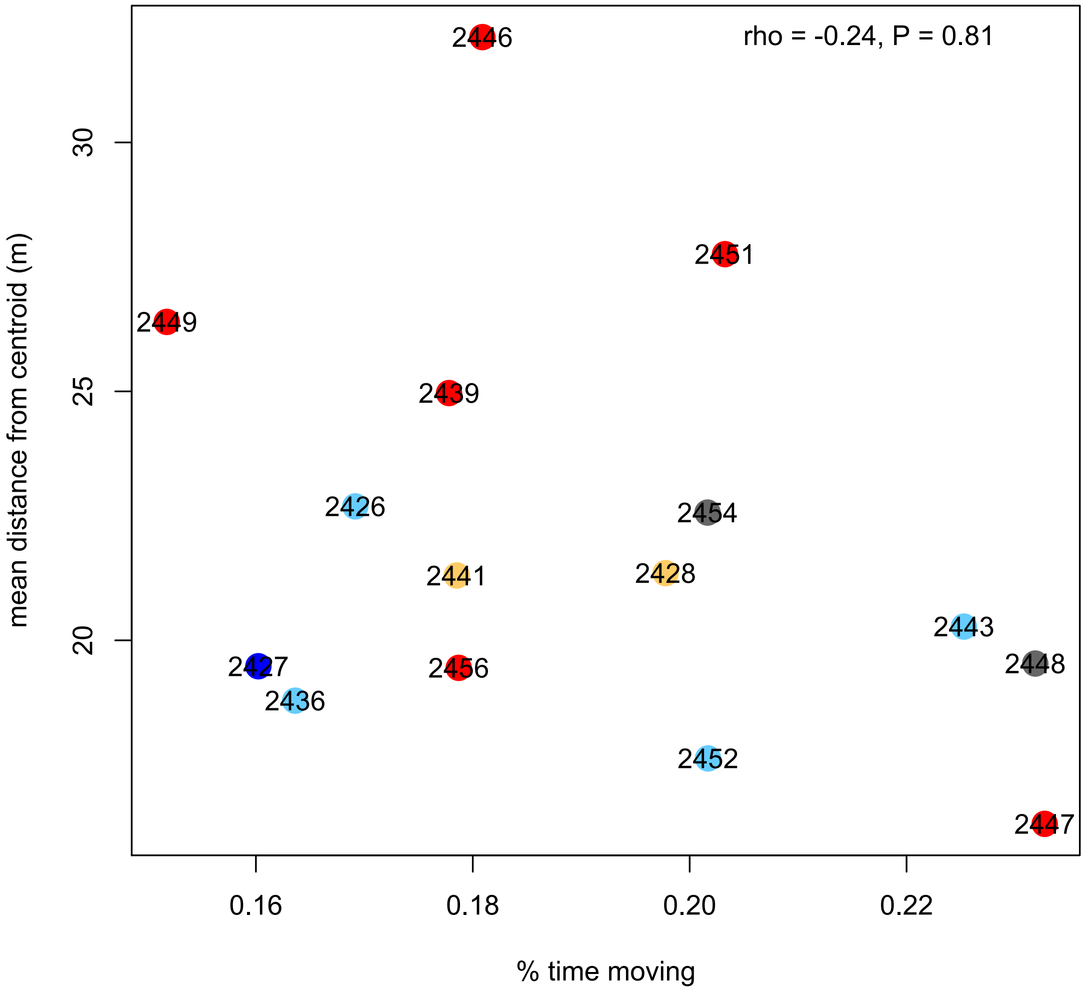

Figure S7: Relationship between percent time moving and individuals' mean distance from the group centroid. Individuals that are more active could end up as being peripheral as their movements more often lead them towards the edge of the group. However, we find no evidence that this relationship exists.

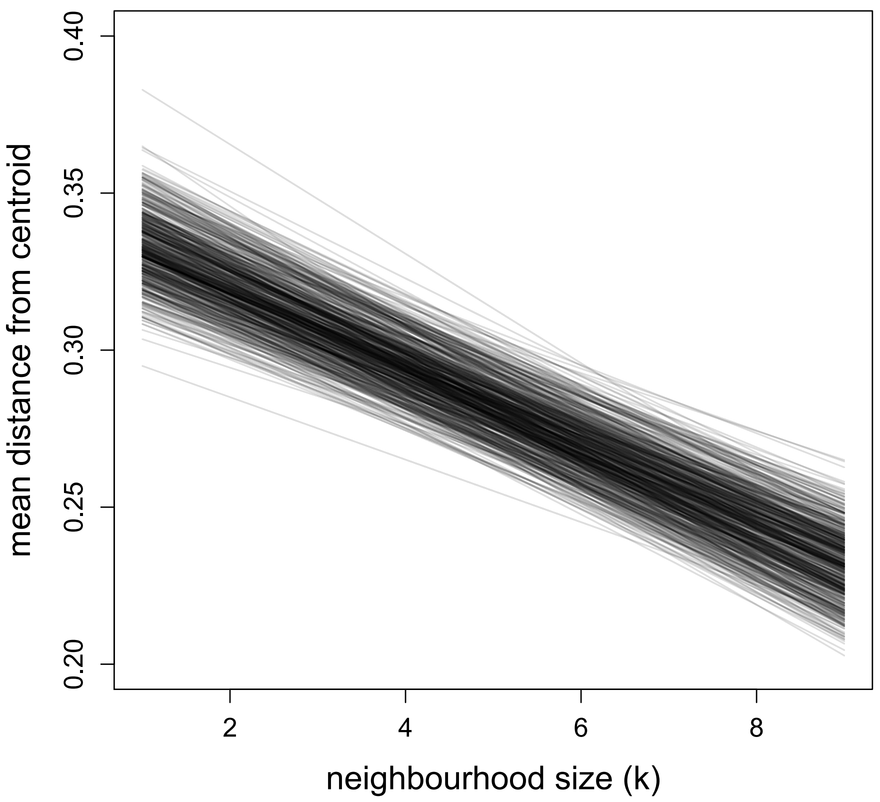

Figure S8. Results from a two-dimensional toy movement model illustrate that individual differences in neighbourhood size can lead to differences in position relative to the group centroid. Parameters used: p = 0.5, s = 0.1, σ = 0.1, t = 1000.

SUPPLEMENTAL TABLES

Table S1. Collar ID, age/sex class and tracking details for GPS-collared baboons

Collar Number	*Sex*	*Age*	*Battery*	*Capture Date*	*Tracking Start Date*	*Tracking End Date*	*Days Tracked*
2426	Male	Sub-adult	D	7/21/2012	8/1/2012	8/31/2012	31
2427	Male	Adult	D	7/21/2012	8/1/2012	9/4/2012	35
2428	Female	Sub-adult	C	7/29/2012	8/1/2012	8/15/2012	15
2430	Female	Adult	D	7/28/2012	8/1/2012	8/3/2012	3
2432	Male	Sub-adult	C	7/22/2012	8/1/2012	8/5/2012	5
2433	Male	Sub-adult	D	7/22/2012	8/1/2012	8/6/2012	6
2434	Male	Adult	D	7/22/2012	8/1/2012	8/2/2012	2
2436	Male	Sub-adult	D	7/22/2012	8/1/2012	9/2/2012	33
2439	Female	Adult	D	7/21/2012	8/1/2012	9/4/2012	35
2441	Female	Sub-adult	C	7/22/2012	8/1/2012	8/29/2012	29
2443	Male	Sub-adult	D	7/23/2012	8/1/2012	9/2/2012	33
2446	Female	Adult	D	7/25/2012	8/1/2012	9/2/2012	33
2447	Female	Adult	D	7/24/2012	8/1/2012	8/31/2012	31
2448	Male	Juvenile	C	7/22/2012	8/1/2012	8/17/2012	17
2449	Female	Adult	D	7/23/2012	8/1/2012	8/31/2012	31
2450	Female	Sub-adult	D	7/24/2012	8/1/2012	8/5/2012	5
2451	Female	Adult	D	7/22/2012	8/1/2012	9/2/2012	33
2452	Male	Sub-adult	C	7/21/2012	8/1/2012	8/14/2012	14
2453	Female	Adult	D	7/25/2012	8/1/2012	8/3/2012	3
2454	Male	Juvenile	C	7/22/2012	8/1/2012	8/14/2012	14
2455	Female	Sub-adult	D	7/28/2012	8/1/2012	8/8/2012	8
2456	Female	Adult	D	7/22/2012	8/1/2012	8/31/2012	31
2457	Male	Adult	D	7/21/2012	8/1/2012	9/4/2012	35
2458	Female	Adult	D	7/22/2012	8/1/2012	8/2/2012	1
2460	Female	Adult	D	7/28/2012	8/1/2012	8/2/2012	1

Table S2. Distance from centroid vs. Identity only – Overall result

Fixed effects

Coefficient	*β ± SE*
Intercept	0.02 ± 0.07

Random effects

Groups	*Variance ± SE*
ID	0.12 ± 0.35
Residual	0.83 ± 0.92

Random effect coefficients

ID	*Intercept*
2426	-0.23
2427	-0.02
2428	0.61
2430	0.22
2432	-0.34
2433	-0.27
2434	-0.28
2436	0.01
2439	0.47
2441	-0.18
2443	-0.02
2446	0.85
2447	-0.27
2448	-0.17
2449	-0.02
2450	-0.30
2451	0.33
2452	-0.15
2453	-0.03
2454	-0.49
2455	0.63
2456	-0.06
2457	-0.14
2458	0.48
2460	-0.02

P-value from permutation test: **P < 0.001**

Table S3. Distance from centroid vs. Age / Sex Class – Overall result

Fixed effects

Coefficient	*β ± SE*
Intercept	0.13 ± 0.20
Adult Male (Reference)	------ 0 ------
Sub-adult Male	-0.34 ± 0.24
Adult Female	-0.05 ± 0.22
Sub-adult Female	-0.17 ± 0.26
Juvenile	-0.24 ± 0.31

Random effects

Groups	*Variance ± SE*
ID	0.12 ± 0.34
Residual	0.83 ± 0.92

P-value from permutation test: P = 0.555

Table S4. Distance from centroid vs. Age / Sex Class - Pairwise comparisons

	Subadult Male	*Adult Female*	*Subadult Female*
Adult Male	-0.34 ± 0.18 **(P = 0.043)**	-0.05 ± 0.30 (P = 0.430)	-0.17 ± 0.21 (P = 0.295)
Subadult Male		0.39 ± 0.19 **(P = 0.020)**	0.17 ± 0.06 **(P = 0.026)**
Adult Female			-0.21 ± 0.23 (P = 0.178)

Note: positive coefficient values indicate that the age-sex class in the row was closer to the group center than the age-sex class in the column.

Table S5. Distance toward front vs. Identity only – Overall result

Fixed effects

Coefficient	*β ± SE*
Intercept	-0.04 ± 0.04

Random effects

Groups	*Variance ± SE*
ID	0.04 ± 0.21
Residual	0.92 ± 0.96

Random effect coefficients

ID	*Intercept*
2426	0.05
2427	0.13
2428	0.08
2430	0.19
2432	-0.04
2433	0.07
2434	-0.10
2436	0.13
2439	0.40
2441	-0.01
2443	0.07
2446	-0.27
2447	-0.25
2448	-0.12
2449	-0.33
2450	-0.13
2451	-0.06
2452	-0.06
2453	-0.17
2454	0.15
2455	-0.10
2456	0.04
2457	0.22
2458	-0.61
2460	-0.26

P-value from permutation test: **P < 0.001**

Table S6. Distance toward front vs. Age / Sex Class – Overall result

Fixed effects

Coefficient	*β ± SE*
Intercept	0.09 ± 0.12
Adult Male (Reference)	------ 0 ------
Sub-adult Male	-0.16 ± 0.15
Adult Female	-0.10 ± 0.14
Sub-adult Female	-0.27 ± 0.16
Juvenile	-0.06 ± 0.20

P-value from permutation test: P = 0.424

Table S7. Distance toward front vs. Age / Sex Class - Pairwise comparisons

	Subadult Male	*Adult Female*	*Subadult Female*
Adult Male	-0.16 ± 0.08 **(P = 0.043)**	-0.10 ± 0.17 (P = 0.701)	-0.27 ± 0.10 **(P = 0.012)**
Subadult Male		0.06 ± 0.12 (P = 0.326)	-0.11 ± 0.09 (P = 0.130)
Adult Female			-0.17 ± 0.16 (P = 0.164)

Note: negative coefficient values indicate that the age-sex class in the row was farther toward the front of the group than the age-sex class in the column.

Table S8. Distance from centroid vs. Dominance rank

Fixed effects

Coefficient	*β ± SE*
Intercept	-0.20 ± 0.12
Dominance rank	0.04 ± 0.01

Random effects

Groups	*Variance ± SE*
Age-sex class	0.06 ± 0.24
Residual	0.93 ± 0.97

P-value from permutation test: **P = 0.048**

Note: a positive coefficient associated with dominance rank indicates that subordinate individuals are farther from the troop center than dominants.

Table S9. Distance toward front vs. Dominance rank

Fixed effects

Coefficient	*β ± SE*
Intercept	0.04 ± 0.07
Dominance rank	-0.01 ± 0.001

Random effects

Groups	*Variance ± SE*
Age-sex class	0.12 ± 0.14
Residual	0.95 ± 0.97

P-value from permutation test: P = 0.106

A negative coefficient associated with dominance rank indicates that dominant individuals are more likely to be found toward the front than subordinates.
